# Reversible shifts between interstitial and epibenthic habitats in evolutionary history: Molecular phylogeny of the marine flatworm family Boniniidae (Platyhelminthes: Polycladida: Cotylea) with descriptions of two new species

**DOI:** 10.1371/journal.pone.0276847

**Published:** 2022-11-23

**Authors:** Aoi Tsuyuki, Yuki Oya, Hiroshi Kajihara

**Affiliations:** 1 Graduate School of Science, Hokkaido University, Sapporo, Hokkaido, Japan; 2 College of Arts and Sciences, J. F. Oberlin University, Machida, Tokyo, Japan; 3 Faculty of Science, Hokkaido University, Sapporo, Hokkaido, Japan; Laboratoire de Biologie du Développement de Villefranche-sur-Mer, FRANCE

## Abstract

Tiny animals in various metazoan phyla inhabit the interstices between sand and/or gravel grains, and adaptive traits in their body plan, such as simplification and size reduction, have attracted research attention. Several possible explanations of how such animals colonized interstitial habitats have been proposed, but their adaptation to this environment has generally been regarded as irreversible. However, the actual evolutionary transitions are not well understood in almost all taxa. In the present study, we show reversible evolutionary shifts from interstitial to epibenthic habitats in the lineage of the polyclad flatworm genus *Boninia*. In addition, we establish two new species of this genus found from different microhabitats on a single beach in Okinawa Island, Japan: (*i*) the interstitial species *Boninia uru* sp. nov. from gravelly sediments and (*ii*) the epibenthic species *Boninia yambarensis* sp. nov. from rock undersurfaces. Our observations suggest that rigid microhabitat segregation exists between these two species. Molecular phylogenetic analyses based on the partial 18S and 28S rDNA sequences of the new *Boninia* species and four other congeners, for which molecular sequences were available in public databases [*Boninia antillara* (epibenthic), *Boninia divae* (epibenthic), *Boninia neotethydis* (interstitial), and an unidentified *Boninia* sp. (habitat indeterminate)], revealed that the two interstitial species (*B*. *neotethydis* and *B*. *uru* sp. nov.) were not monophyletic among the three epibenthic species. According to ancestral state reconstruction analysis, the last common ancestor of the analyzed *Boninia* species inhabited interstitial realms, and a shift to the epibenthic environment occurred at least once. Such an “interstitial to noninterstitial” evolutionary route seems to be rare among Animalia; to date, it has been reported only in acochlidian slugs in the clade Hedylopsacea. Our phylogenetic tree also showed that the sympatric *B*. *uru* sp. nov. and *B*. *yambarensis* sp. nov. were not in a sister relationship, indicating that they colonized the same beach independently rather than descended *in situ* from a common ancestor that migrated and settled at the beach.

## Introduction

Animals inhabiting the space between sand and/or gravel grains have attracted the attention of biologists since the 1930s [cf. 1], primarily due to their miniaturized body size [e.g., 2–6] and ecological importance [e.g., 7], although the existence of such tiny animals was recognized by zoologists in the 19th century [e.g., 8, 9]. An assemblage of such animals is known as interstitial fauna, a term first introduced in 1935 to refer to small copepods, nematodes, rotifers, and protozoans found at sandy beaches [10]. In addition, the term mesopsammon (literally meaning “between sand”) has been used since the 1940s, initially and chiefly in German literature [11]. Other terms, such as meiobenthos and meiofauna, are also often used interchangeably with interstitial fauna. Technically, however, meiobenthos (or meiofauna) are organisms that can pass through a 1-mm mesh but are retained by a 45-μm mesh. Therefore, interstitial or mesopsammic animals are not necessarily meiobenthic in size.

To date, interstitial animals have been documented in at least 23 of the ~34 currently recognized metazoan phyla [12], and different evolutionary scenarios have been proposed to explain the existence of such animals [e.g., 4]. Recent phylogenetic studies have shown that some annelid interstitial taxa had independently derived from larger epibenthic ancestors either by progenesis or stepwise miniaturization depending on the taxa [6, 13]. Additional research on these evolutionary processes has shed light on other taxa, including Enteropneusta (Hemichordata) [14], Acochlidiacea (Heterobranchia: Gastropoda) [15], Rhodopemorpha (Heterobranchia: Gastropoda) [16], and Ostracoda [17]; however, the vast majority of relevant animal groups have yet to be studied in this context, including the phylum Platyhelminthes and one of its constituent subtaxa, the order Polycladida.

According to recent phylogenomic analyses [18, 19], Polycladida is reciprocally monophyletic to another order, Prorhynchida, which together form a clade that is sister to the remaining platyhelminths, excluding Catenulida and Macrostomorpha. All the known catenulids and macrostomorphans are microscopic, and some species live in interstitial habitats [20], whereas some almost exclusively mesopsammic flatworm groups, such as Proseriata and Rhabdocoela, are more deeply nested in the phylum [18, 19].

Little attention has hitherto been paid to interstitial polyclads, probably due to their rarity. Indeed, polyclads were not mentioned in Swedmark’s 1964 seminal work on marine interstitial fauna [21]. Polyclads are mostly free-living marine flatworms that are categorized into two suborders: Acotylea (with 29 families [22]) and Cotylea (with 12 families [cf. 23]). Most polyclads have a relatively large body size (≥5 mm) and dwell on the surface of the marine bottom [24], whereas only a tiny fraction inhabit interstitial environments [25]; of the ~800–1,000 species of polyclads described worldwide to date, 12 (5 in 4 acotylean families and 7 in 3 cotylean families) are mesopsammic in the adult stage [25–30]. On the other hand, some surface dwellers can also be found from interstitial environments when they are juveniles or subadults (authors’ personal observation). In this paper, we restrict the notion of interstitial polyclads to refer to those that inhabit sand/gravel sediments even after reaching sexual maturity. Likewise, epibenthic and noninterstitial species refer to the ones inhabiting environments other than the interstitial one, such as undersurfaces of rocks, when they are fully mature, while juveniles or subadults may dwell in sand or gravel.

The cotylean polyclad family Boniniidae Bock, 1923 [31] is interesting in terms of the evolutionary shift between noninterstitial and interstitial habitats because it harbors members that live in both environments. However, the phylogenetic inter-relationships within this family are yet to be resolved because of insufficient taxon sampling [23]. Currently, seven named species of boniniids are classified into three genera: *Boninia* Bock, 1923 [31] (5 species), *Paraboninia* Prudhoe, 1944 [32] (1 species), and *Traunfelsia* Laidlaw, 1906 [33] (1 species). To date, only *Boninia neotethydis* Curini-Galletti & Campus, 2007 [30] from the Mediterranean and Red Sea has been described as a permanent interstitial representative based on adult specimens [30]. Boniniids are morphologically characterized by having (*i*) a narrow and elongate body with a pair of pointed tentacles located at the anterolateral margins, (*ii*) a male copulatory apparatus that includes an unarmed penis papilla and one or several prostatoid organ(s) with stylets, (*iii*) a female copulatory apparatus with a Lang’s vesicle, and (*iv*) a ventral adhesive organ located at the posterior end of the body [24].

The genus *Boninia* contains *Boninia antillara* (Hyman, 1955) [34]; *Boninia divae* Du Bois-Reymond Marcus & Marcus, 1968 [35]; *Boninia mirabilis* Bock, 1923 [31]; *Boninia neotethydis*; and *Boninia oaxaquensis* Ramos-Sánchez et al., 2020 [36]. Morphologically, these species are distinguishable from the other two boniniids, namely *Traunfelsia elongata* Laidlaw, 1906 [33] and *Paraboninia caymanensis* Prudhoe, 1944 [32], by having one or more girdle(s) of prostatoid organs opening into the male atrium [37]. Except *B*. *neotethydis*, the abovenamed *Boninia* species have been reported as dwellers on undersurfaces of the rocks in fully mature state [31, 34–36].

During our polyclad faunal survey in Japan, we found two undescribed species of *Boninia* on a single beach, with one species collected from an interstitial environment and the other from rock undersurfaces. From these findings, we hypothesized some evolutionary scenarios pertaining to (*i*) shifts between interstitial and noninterstitial microhabitats and (*ii*) settlement of the two species at the same beach. Of the conceivable hypothetical scenarios, one suggests the two interstitial species of *Boninia* (*B*. *neotethydis* in the Mediterranean/Red Sea and the undescribed form in Japan) being exclusively monophyletic. This “interstitial monophyly hypothesis” would be supported if adaptation from a noninterstitial to interstitial lifestyle was evolutionarily irreversible and uncommon. In another hypothesis, the last common ancestor of our two new species, which could be either interstitial or noninterstitial, settled at the beach, and one of the two species subsequently changed microhabitat. This “*in situ* speciation hypothesis” would be favored if such an event was considered rare that the settlement of two closely related species (i.e., in the same genus) at a single beach happened twice independently.

Overall, the aims of this study were to (*i*) provide formal taxonomic descriptions of the two new *Boninia* species and (*ii*) test the abovementioned hypotheses using ancestral state reconstruction analysis based on a molecular phylogenetic tree of Boniniidae.

## Materials and methods

### Ethics statement

No permissions were required for collecting materials in this study. Our sampling locality was not privately owned but open to the public. We did not involve endangered or protected species.

### Collection of specimens and morphological observations

Specimens were collected at Nagahama Beach, Okinawa Island, Japan. Gravelly sediment samples near the edge of the water were agitated in tap water to extract animals. The supernatant was filtered using an about 1-mm meshed dip net, and the residue was subsequently transferred into seawater. In total, six polyclads were extracted from the sediment samples. Other six polyclads crawling on undersurfaces of rocks were also collected at the sandy beach in the intertidal area. All flatworm specimens were anesthetized in a MgCl_2_ solution prepared with tap water to match the seawater salinity using an IS/Mill-E refractometer (AS ONE, Japan), after which they were photographed using a Nikon D5600 digital camera with external strobe lightning provided by a pair of Morris Hikaru Komachi Di flash units. Each polyclad specimen was fixed and preserved using one of the four protocols shown in Table 1.

**Table 1:** List of protocols for fixation and GenBank accession numbers of the sequences of materials.

Species	ICHUM number	Protocol for fixation	Type status	Sequence data		
				COI	18S	28S
*Boninia uru* sp. nov.	ICHUM 8278	i	holotype	LC699268	—	LC699275
*Boninia uru* sp. nov.	ICHUM 8279	ii	paratype	—	—	—
*Boninia uru* sp. nov.	ICHUM 8280	ii	paratype	—	—	—
*Boninia uru* sp. nov.	ICHUM 8281	iii	paratype	LC699269	LC699274	LC699276
*Boninia uru* sp. nov.	ICHUM 8282	iii	paratype	LC699270	—	LC699277
*Boninia uru* sp. nov.	ICHUM 8283	ii	paratype	—	—	—
*Boninia yambarensis* sp. nov.	ICHUM 8284	i	holotype	LC699271	LC699273	LC699278
*Boninia yambarensis* sp. nov.	ICHUM 8285	i	paratype	—	—	LC699279
*Boninia yambarensis* sp. nov.	ICHUM 8286	i	paratype	—	—	LC699280
*Boninia yambarensis* sp. nov.	ICHUM 8287	i	paratype	LC699272	—	LC699281
*Boninia yambarensis* sp. nov.	ICHUM 8288	i	paratype	—	—	LC699282
*Boninia yambarensis* sp. nov.	ICHUM 8289	iv	paratype	—	—	—

The fixation protocols (i–iv) are: (i) a part of the body was removed and preserved in 99.5% ethanol for DNA extraction and the rest of the body was fixed in Bouin’s solution for 24 h and subsequently preserved in 70% ethanol; (ii) the whole body was fixed in Bouin’s solution for 24 h and subsequently preserved in 70% ethanol; (iii) the whole body was preserved in 99.5% ethanol for DNA extraction; and (iv) the whole body was mounted on a glass slide, squeezed under a cover slip, and preserved in 10% formaldehyde solution in seawater.

For histological examination, tissues were prestained with acid fuchsin, dehydrated in an ethanol series, cleared in xylene, embedded in paraffin wax, and sectioned serially at a thickness of 4 μm using a microtome. Sections were stained using either hematoxylin and eosin (HE) or Mallory’s trichrome (MT) methods, mounted on glass slides, and then embedded in Entellan New (Merck, Germany) under cover slips. They were then observed and photographed using a Nikon D5300/5600 digital camera under an Olympus BX51 compound microscope.

Type specimens have been deposited in the Invertebrate Collection of the Hokkaido University Museum (ICHUM). All graphical treatments were completed using Adobe Photoshop CC. Illustrations were prepared using Adobe Illustrator CC.

### DNA extraction, PCR, and sequencing

Total DNA was extracted using a DNeasy Blood & Tissue Kit (Qiagen, Germany) after specimens were kept overnight at 55°C in 180 μl of ATL buffer (Qiagen, Germany) with 20 μl of proteinase K (>700 U/ml; Kanto Chemical, Japan). As a reference for DNA barcoding, a partial sequence (709 bp) of the cytochrome *c* oxidase subunit I (COI) gene was determined. For phylogenetic inference, fragments of the 18S rDNA (18S; 1,758 bp) and 28S rDNA (28S; 1,014–1,015 bp) were sequenced. Amplification of the three gene markers was performed using polymerase chain reaction (PCR) via a 2720 Thermal Cycler (Applied Biosystems, USA); 10-μl reaction volumes were used, each of which contained 1 μl of total DNA template, 1 μl of 10 × ExTaq buffer (Takara Bio, Japan), 2 mM of each dNTP, 1 μM of each primer, and 0.25 U of Takara Ex Taq DNA polymerase (5 U/μl; Takara Bio, Japan) in deionized water. The forward and reverse primer pairs listed in Table 2 were used. The PCR amplification conditions were as follows: 94°C for 5 min; 35 cycles of 94°C for 30 s, 50°C (18S and COI) or 52.5°C (28S) for 30 s, and 72°C for 2 min (18S), 1.5 min (28S), or 1 min (COI); and 72°C for 7 min. PCR products were purified enzymatically using ExoSAP-IT reagent. All nucleotide sequences were determined using direct sequencing with a BigDye Terminator Kit ver. 3.1 and a 3730 Genetic Analyzer (Life Technologies, California, USA) with the primers listed in Table 2. Sequences were checked and edited using MEGA ver. 7.0 [38]. All edited sequences have been deposited in DDBJ/EMBL/GenBank with accession numbers LC699268–LC699282 (Table 1).

**Table 2:** List of primers used in this study.

Gene	Primer name	Sequence	Application	Reference
COI	Acotylea_COI_F	ACTTTATTCTACTAATCATAAGGATATAGG	amplification and sequencing	[39]
COI	Acotylea_COI_R	CTTTCCTCTATAAAATGTTACTATTTGAGA	amplification and sequencing	[39]
18S	hrms18S_F	ATCCTGCCAGTAGTCATATGC	amplification and sequencing	[22]
18S	hrms18S_Fi1	GCCGCGGTAATTCCAGCTCC	sequencing	[22]
18S	hrms18S_Fi2	GGGTTCCGGGGGAAGTATG	amplification and sequencing	[22]
18S	hrms18S_R	CTACGGAAACCTTGTTACGAC	amplification and sequencing	[22]
18S	hrms18S_Ri1	CTTTAATATACGCTATTGGAGCTGG	sequencing	[22]
18S	hrms18S_Ri2	CTATTTAGTGGCTAGAGTCTCGTTCG	sequencing	[22]
28S	fw1	AGCGGAGGAAAAGAAACTA	amplification and sequencing	[40]
28S	hrms_fw2	AGAAGTACCGCGAGGGAARGTTG	sequencing	[22]
28S	rev4	GTTAGACTYCTTGGTCCGTG	sequencing	[40]
28S	rev2	ACGATCGATTTGCACGTCAG	amplification and sequencing	[40]

### Phylogenetic analyses

For phylogenetic analyses, a concatenated dataset (2,685 bp) comprising partial 18S (1,739 bp) and 28S rDNA (946 bp) sequences was prepared. Additional 18S and 28S rDNA sequences of four species from Boniniidae, which were available in a public database, were downloaded from GenBank (Table 3). To assess the last common ancestral state of boniniids, its proposed sister groups Amyellidae Faubel, 1984 [37] and Theamatidae Marcus, 1949 [23, 26, 41] were also included in the analysis (Table 2). The three cotylean species *Cestoplana rubrocincta* (Grube, 1840) [42], *Pericelis flavomarginata* Tsuyuki et al., 2020 [43], and *Pericelis tectivorum* Dittmann et al., 2019 [44] were used as outgroups (Table 2). Sequences were aligned using MAFFT ver. 7.427 [45], with the L-INS-i strategy selected using the “Auto” option. Ambiguous sites were trimmed using Clipkit ver. 1.0 via the “kpic” option [46]. The optimal substitution models, selected using PartitionFinder ver. 2.1.1 [47] according to the Akaike Information Criterion [48] with the greedy algorithm [49], were GTR+I+G for both the 18S and 28S partitions. Phylogenetic analysis was performed using the maximum likelihood (ML) method via RAxML ver. 8.2.10 [50]. Bayesian inference (BI) of the phylogeny was performed using MrBayes ver. 3.2.3 [51, 52] with two independent runs of Metropolis-coupled Markov chain Monte Carlo (MCMC), each consisting of four chains of 2,000,000 generations. All parameters (*statefreq*, *revmat*, *shape*, and *pinvar*) were unlinked between each position; trees were sampled every 100 generations. The first 25% of the trees were discarded as burn-in before a 50% majority-rule consensus tree was constructed. Convergence was confirmed using an average standard deviation of split frequencies of 0.001989, potential scale reduction factors for all parameters of 1.000–1.002 and effective sample sizes for all parameters of >209. Nodal support within the ML tree was assessed using analysis of 1,000 bootstrap (BS) pseudoreplicates [53]. ML BS values ≥70% and posterior probability (PP) values ≥90% were considered to indicate clade support (here, combined nodal support is indicated as “PP/BS”).

**Table 3:** List of species used for the molecular phylogenetic analysis, and their respective collection localities and habitats, GenBank accession numbers, and references.

Species	Collection locality	Habitat	GenBank accession		Reference
			18S rDNA	28S rDNA	
Amyellidae					
*Chromyella* sp.	Bocas del Toro, Panama	interstitial (sandy sediments)	KC869795	KC869848	[54]; Laumer pers. comm.
Boniniidae					
*Boninia uru* sp. nov.	Okinawa Island, Okinawa, Japan	interstitial (among course gravelly sediments)	LC699274	LC699276	this study
*Boninia antillara*	Great Lameshure Bay, St. John, US Virgin Islands	epibenthic (under rocks)	—	MH700282	[23]
*Boninia divae*^a^	Playa Santa Cruz, Curaçao	epibenthic (under rocks)	—	MH700280	[23]
*Boninia neotethydis*	Eilat, Israel	interstitial (course sediments)	—	MH700283	[23]
*Boninia yambarensis* sp. nov.	Okinawa Island, Okinawa, Japan	epibenthic (under rocks)	LC699273	LC699278	this study
*Boninia* sp.^b^	Bocas del Toro, Panama	—^c^	KC869793	KC869846	[54]; Laumer pers. comm.
Theamatidae					
*Theama mediterranea*	Rovinj, Croatia	interstitial (sandy sediments)	—	MN384705	[41]
*Theama* sp.	Bocas del Toro, Panama	interstitial (sandy sediments)	KC869792	KC869845	[54]; Laumer pers. comm.
Outgroup					
*Cestoplana rubrocincta*	Naples, Italy	epibenthic (under rocks)	MN384689	MN334198	[41]
*Pericelis flavomarginata*	Kagoshima, Japan	epibenthic (under rocks)	LC672041	LC568535	[43, 55]
*Pericelis tectivorum*	Aquaria Innsbruck, Austria	epibenthic (under rocks or other objects)	MK181525	MK181524	[41, 44]

^a^The specimen is currently registered as *B*. *antillara* based on the taxon concept of Litvaitis et al. [23] in that *B*. *divae* should be synonymized with *B*. *antillara*, but it was originally identified as *B*. *divae* based on the morphology [23].

^b^In the GenBank database, the specimen was assigned to *Boninia divae*, but it should be “*Boninia* sp.” because it was unidentifiable due to its juvenile state (cf. https://mczbase.mcz.harvard.edu/guid/MCZ:IZ:132897).

^c^Although the specimen was collected from an interstitial habitat, we treated the habitat of *Boninia* sp. as indeterminate in this paper because we cannot evaluate the habitat in adult state due to its juvenile state (see Introduction).

### Ancestral state reconstruction related to microhabitat

The habitat of each ingroup species was determined from the original description (Table 3). The habitat information of the three unidentified species, *Boninia* sp., *Chromyella* sp., and *Theama* sp., was provided directly by the collector, Christopher Edward Laumer (Table 3). The possible ancestral states were reconstructed using Bayesian Binary MCMC (BBM) analysis implemented in RASP 4.2 [56, 57]. To take phylogenetic uncertainty into account, 10 trees randomly selected from the post burn-in trees generated by MrBayes ver 3.2.3 were used as input trees. BBM analysis was then run on a consensus Bayesian tree. The MCMC chain was run for 50,000 generations using 10 chains and sampled every 100 generations. A fixed (LC) model that did not allow null root distribution was used to conduct the analysis.

### Nomenclatural acts

The electronic vision of this article conforms to the requirements of the amended International Code of Zoological Nomenclature, and hence the new names contained herein are available under that Code from the electronic edition of this article. This published work and the nomenclatural acts it contains have been registered in ZooBank, the online registration system for the ICZN. The ZooBank LSIDs (Life Science Identifiers) can be resolved, and the associated information viewed through any standard web browser by appending the LSID to the prefix “http://zoobank.org/.” The LSID for this publication is: urn:lsid:zoobank.org:pub:B88724EB-7332-419E-A4C4-A1DFC05E121F. The electronic edition of this work was published in a journal with an ISSN and has been archived and is available from the following digital repositories: PubMed Central, LOCKSS, ResearchGate, HUSCAP.

## Results

### Taxonomy

#### Order Polycladida Lang, 1881 [58]

Suborder Cotylea Lang, 1884 [59]

Superfamily Boninioidea Bock, 1923 [31] sensu Dittmann et al. [41]

Family Boniniidae Bock, 1923 [31]

Genus *Boninia* Bock, 1923 [31]

(Type species *Boninia mirabilis* Bock, 1923 [31])

#### *Boninia uru* sp. nov.

urn:lsid:zoobank.org:act:2199A291-F959-4577-B609-E8814BC4C7B5

(Figs 1–3).

**Fig 1:**
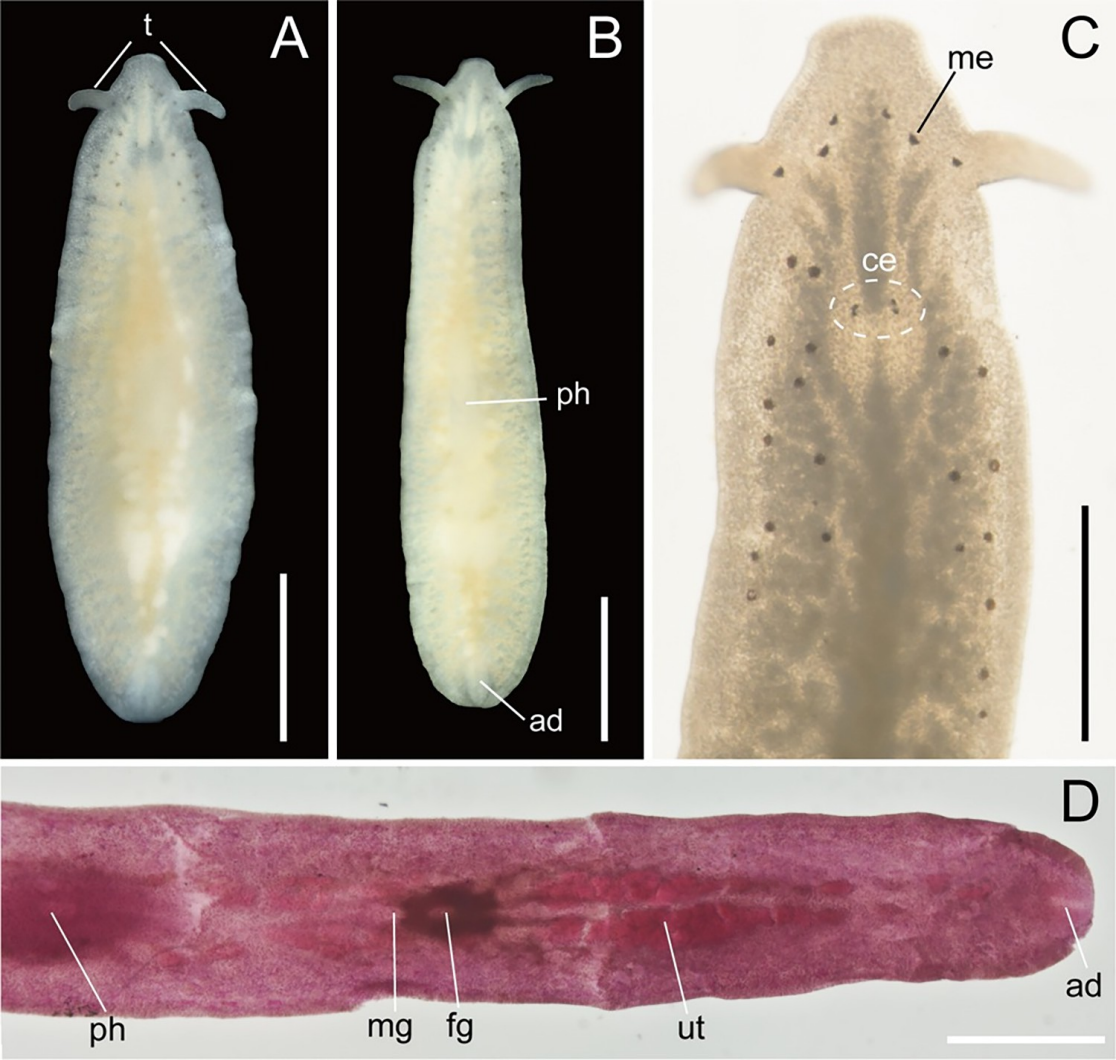
*Boninia uru* sp. nov., ICHUM 8278: Photographs of living and fixed specimens. (A) Entire animal, living state, dorsal view; (B) entire animal, living state, ventral view; (C) magnification of anterior body, living state; (D) posterior body showing reproductive organs, fixed state, stained with acid fuchsin and cleared in xylene, ventral view. Abbreviations: ad, adhesive organ; ce, cerebral eyespots; fg, female gonopore; me, marginal eyespot; mg, male gonopore; ph, pharynx; t, tentacles; ut, uterus. Scale bars: A–C, 1 mm; D, 0.5 mm.

**Fig 2:**
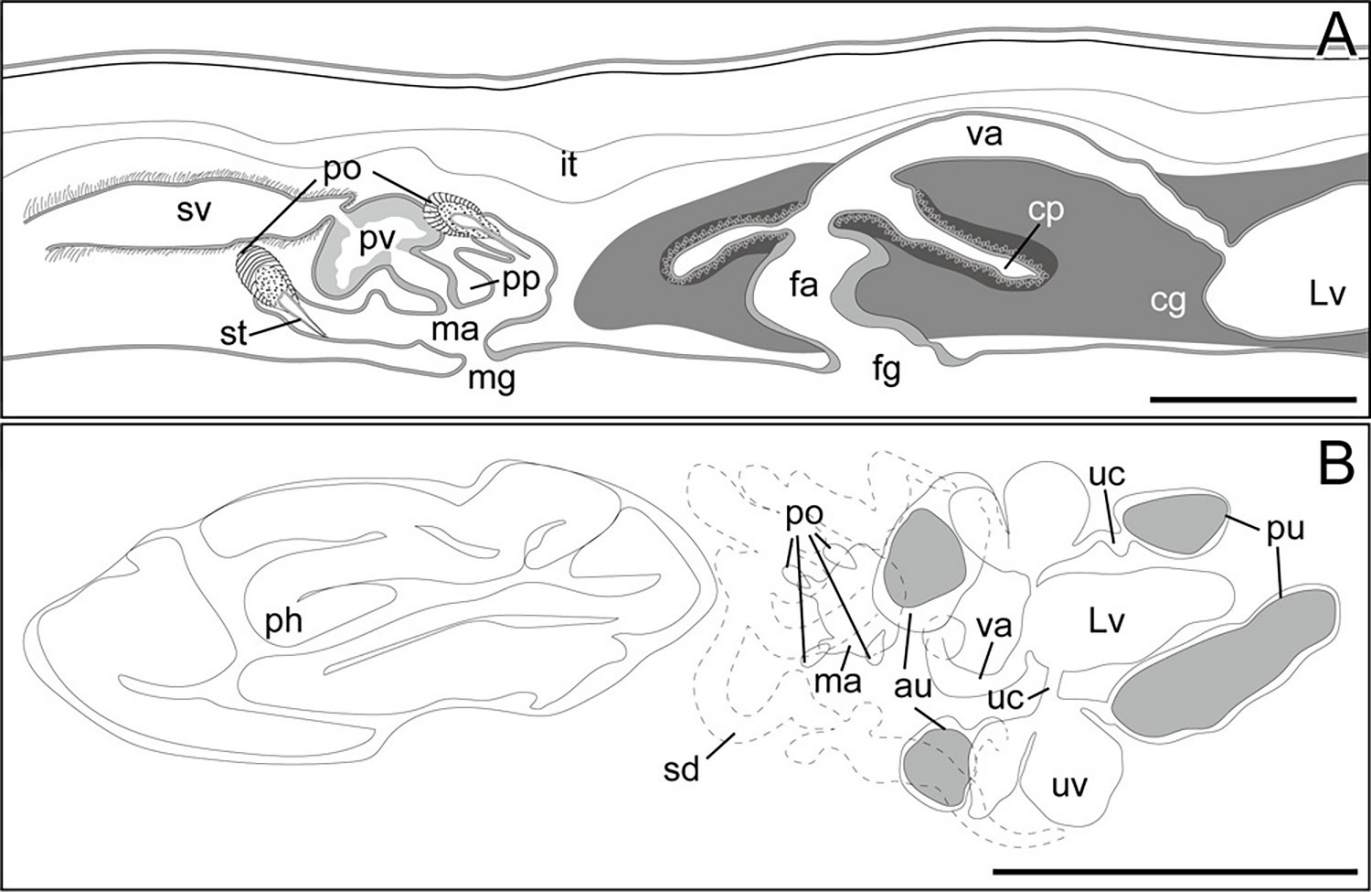
Schematic diagrams of *Boninia uru* sp. nov. (anterior to the left). (A) ICHUM 8278 (holotype), copulatory complex; (B) ICHUM 8283 (paratype), pharynx, male and female copulatory apparatuses. Abbreviations: au, anterior dilations of uteri; cg, cement glands; cp, cement pouch; fa, female atrium; fg, female gonopore; it, intestine; Lv, Lang’s vesicle; ma, male atrium; mg, male gonopore; ph, pharynx; po, prostatoid organ(s); pp, penis papilla; pu, posterior dilations of uteri; pv, prostatoid vesicle; sd, sperm duct; st, stylet; sv, seminal vesicle; uc, uterine canal; uv, uterine vesicle; va, vagina. Scale bars: A, 100 μm; B, 300 μm.

**Fig 3:**
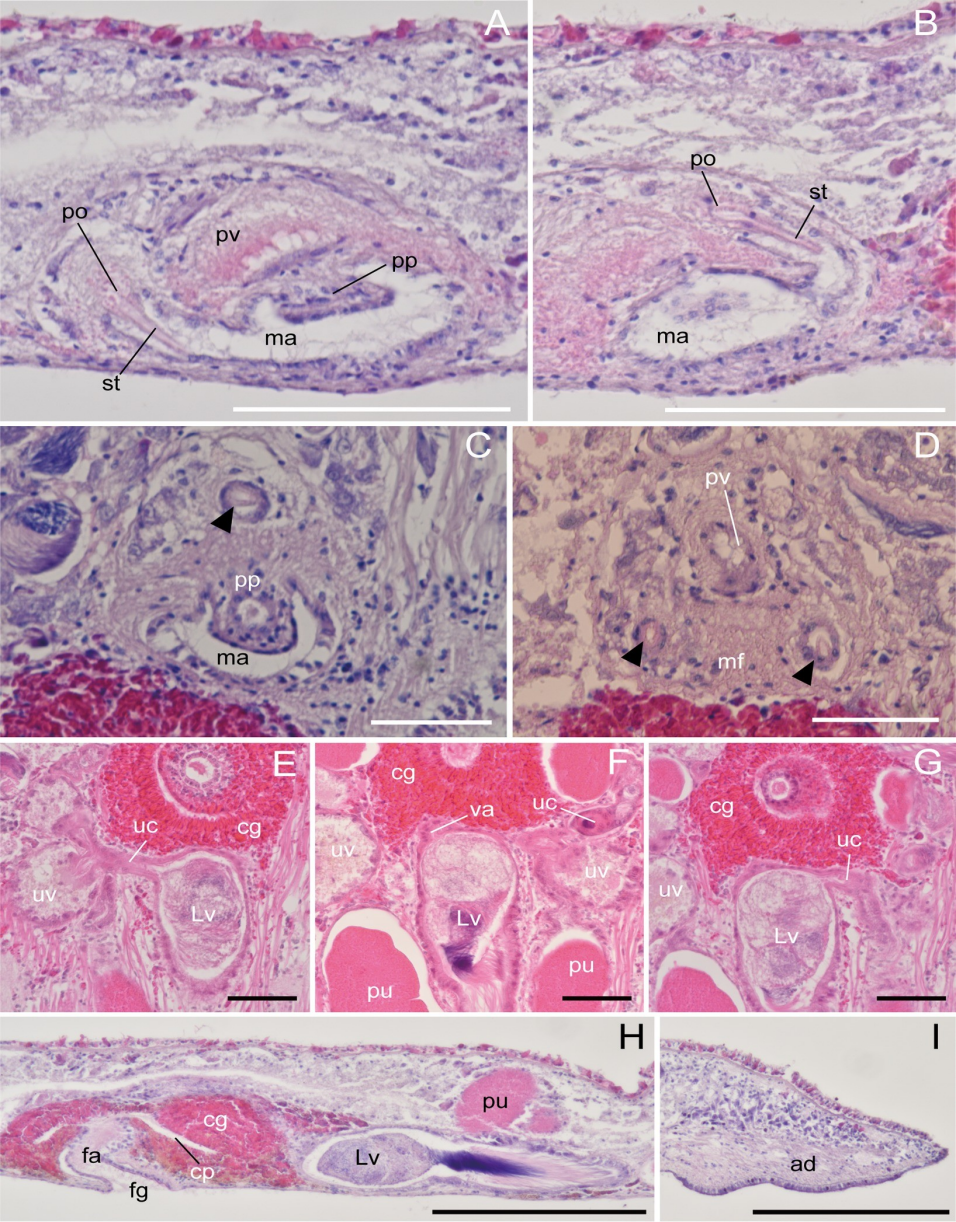
*Boninia uru* sp. nov.: Photomicrographs of sagittal (A, B, H, I) and horizontal (C–G) sections. (A, B) ICHUM 8278 (holotype), male copulatory apparatus, anterior to the left; (C, D) ICHUM 8279 (paratype), male copulatory apparatus, with stylet of the prostatoid organ indicated by arrowheads; (E–G) ICHUM 8283 (paratype), connection between uterine canal and Lang’s vesicle; (H) ICHUM 8278 (holotype), female copulatory complex; (I) ICHUM 8278 (holotype), adhesive organ. Abbreviations: ad, adhesive organ; cg, cement glands; cp, cement pouch; fa, female atrium; fg, female gonopore; Lv, Lang’s vesicle; ma, male atrium; mf, muscle fiber; po, prostatoid organ(s); pp, penis papilla; pu, posterior dilations of uteri; pv, prostatoid vesicle; st, stylet; uc, uterine canal; uv, uterine vesicle. Scale bars: A, B, 100 μm; C–G, 50 μm; H, I, 200 μm.

#### Material examined

*Holotype*. ICHUM 8278, sagittal sections, three slides; Nagahama Beach, Okinawa, Japan (26.6242°N, 128.1843°E); coll. Y. Oya, December 14, 2019.

*Paratypes*. Five specimens (collection site and collector same as holotype). ICHUM 8279, horizontal sections, two slides; December 13, 2019. ICHUM 8280, sagittal sections, two slides; December 14, 2019. ICHUM 8281, unsectioned, preserved in 99.5% ethanol; December 13, 2019. ICHUM 8282, unsectioned, preserved in 99.5% ethanol; December 14, 2019. ICHUM 8283, horizontal sections, three slides; December 14, 2020.

#### Etymology

The specific name *uru*, an Okinawan dialect meaning “coarse sand,” is derived from the habitat of the species.

#### Diagnosis

Body narrow and elongated; one pair of pointed tentacles located at anterolateral margins; four cerebral eyespots and 21–29 marginal eyespots; 2–4 prostatoid organs arranged into single girdle; Lang’s vesicle fully ciliated; subepidermal muscle fibers of adhesive area not well developed.

#### Description

Body elongated, tapered posteriorly, 3.0–4.5-mm long (4.5 mm in holotype) and 0.65–0.90-mm wide (0.72 mm in holotype) in anesthetized living state (Fig 1A and 1B). Pair of pointed tentacles located at sides of head, 0.08–0.38-mm long (0.38 mm in holotype) (Fig 1A–1C). Dorsal surface smooth, translucent, without any coloration (Fig 1A). Ventral surface translucent (Fig 1B).

Tentacular eyespots absent. Pair of two cerebral eyespots (ca. 24 μm in diameter) located at each anterior side of brain; two eyespots in each pair lying close to each other (Fig 1C). Marginal eyespots (ca. 55 μm in diameter), 21–29 in number (27 in holotype), distributed sparsely in anterior quarter of body along margins on both sides (Fig 1A–1C). Diameter of marginal eyespots twice as large as that of cerebral eyespots (Fig 1C).

Intestine highly branched, spreading all over body. Pharynx ruffled, 0.5–1.1-mm long (1.1 mm in holotype), lying on body center (Fig 1B). Mouth situated in center of pharynx.

Male gonopore situated immediately behind pharynx (Fig 1D). Male copulatory apparatus consisting of elongated seminal vesicle, interpolated prostatoid vesicle, penis papilla, and 2–4 prostatoid organs (Fig 2A and 2B). Pair of sperm ducts running on each side of midline, curving at position of posterior end of pharyngeal pouch to separately enter into seminal vesicle (Fig 2B). Seminal vesicle elongated, lined with flat nucleated epithelium, coated with thin muscle fibers, distally opening into prostatoid vesicle (Fig 2A). Prostatoid vesicle lined with high epithelium, connecting to penis papilla (Figs 2A and 3A). Penis papilla unarmed, 20-μm long in dorsoventral axis, projecting into male atrium (Figs 2A and 3A). Inner wall of male atrium well ciliated (Fig 3A and 3B). Two prostatoid organs present, each located anterior and posterior to male atrium in the holotype (ICHUM 8278) (Figs 2A, 3A and 3B) and one of the two sectioned paratypes (ICHUM 8280); three or four prostatoid organs radially arranged around male atrium in the other horizontally sectioned paratypes (ICHUM 8279 and 8283, respectively) (Figs 2B, 3C and 3D); prostatoid organs arranged into single girdle. Each prostatoid organ oval in shape, 50-μm wide, with sclerotized stylet (37 μm in length), protruding into male atrium (Figs 2A and 3A–3D). Extracapsular glands (“prostatoid organ glands”) not well developed (Fig 3A and 3B). Muscle fibers surrounding male atrium, prostatoid organs, and prostatoid vesicle (Fig 3D).

Pair of uterine canals running on both sides of midline, connecting to anterior part of Lang’s vesicle laterally through short branches; each canal with two uterine vesicles (Figs 2B and 3E–3G). Each uterine canal forming uteri at most anterior and posterior dilations; uteri filled with eggs (Figs 2B and 3F). Lang’s vesicle elongated (309 μm in its long axis; 63 μm in its short axis), placed between one pair of posterior uteri; inner wall lined with cilia; elongated cilia observed in posterior region (Fig 3H). Vagina ciliated, leading from Lang’s vesicle to cement pouch (Fig 2A and 2B). Cement glands numerous, concentrated around female copulatory apparatus and releasing contents into cement pouch (Figs 2A and 3E–3H).

Epidermis on dorsal side ciliated, with numerous ovoid rhabdites (Fig 3A and 3B). Ventral epidermis ciliated except for adhesive area (Fig 3A and 3B).

Adhesive organ located at posterior end of body on ventral side (Figs 1B, 1D and 3I). Subepidermal muscle fibers not well developed in adhesive area, surface of which are covered by thick glandular epithelium (Fig 3I).

#### Distribution

To date, known only from the type locality: Nagahama Beach, eastern coast of Okinawa Island, Japan.

#### Habitat

To date, confirmed only from gravelly habitats in intertidal coarse sediments.

#### Remarks

Our specimens are assigned to *Boninia* because they conform to the generic diagnosis of Curini-Galletti & Campus [30], i.e., they have two or more prostatoid organs with stylets opening into the male atrium. *Boninia uru* sp. nov. can be easily distinguished from *B*. *antillara*, *B*. *divae*, and *B*. *mirabilis* by its single girdle of prostatoid organs [30, 31] (Table 4). The other two congeners *B*. *neotethydis* and *B*. *oaxaquensis* have a single girdle of prostatoid organs, as in the new species; however, *B*. *uru* sp. nov. is distinguishable from these two species by its small number (2–4) of prostatoid organs (10–18 organs in *B*. *neotethydis* and 16–24 organs in *B*. *oaxaquensis*). Additionally, the arrangement of eyespots enable discrimination between *B*. *uru* sp. nov. and its congeners. The new species and *B*. *neotethydis* are distinguished from the other species of *Boninia* by having just four cerebral eyespots (Table 4), and they are distinguished from each other in terms of the number of marginal eyespots (21–29 in *B*. *uru* sp. nov.; 6–16 in *B*. *neotethydis*). Also, the uterine canal connecting lateral to Lang’s vesicle of *B*. *uru* sp. nov. is peculiar among the known *Boninia* species except for *B*. *oaxaquensis* (the relevant morphology is unknown) (Table 4).

**Table 4:** Comarison of selected characters among *Boninia* species.

Species	*B*. *antillara*	*B*. *divae*	*B*. *mirabilis*	*B*. *neotethydis*	*B*. *oaxaquensis*	*B*. *uru* sp. nov.	*B*. *yambarensis* sp. nov.	*Boninia* sp.
Body size Length = L Width = W	8 mm (L), 2 mm (W)^a^; 16 mm (L), 3.3, 3.5, and 3.9 mm (W)^b^	30–50 mm (L), 2–4 mm (W)^d^	29 mm (L), 4.5 mm (W) (preserved state)	60 mm (L), 5 mm (W)	3–11 mm (L), 1–3 mm (W)	3–4.5 mm (L); 0.65–0.9 mm (W)	13.9–22.4 mm (L); 0.93–1.25 mm (W)	?
**Marginal eyespot number**	about 40^a^	numerous^d^	numerous, present dorsally and ventrally	10–18 (3–8 per side)	36–126	21–29, only dorsally	19–42, only dorsally	?
**Cerebral eyespot number**	ca. 30^a^	numerous (arranged in two long bands)^d^	13 (arranged in 2 separate clusters)	4 (2 pairs, each pair lying close to each other)	14–66	4 (two pairs, each pair lying close to each other)	6–7 (3–4 pairs; 2 eyespots lying close to each other whereas 1 or 2 eyespots located posteriorly)	?
Relative diameter of eyespots Marginal eyespot: M Cerebral eyespots: C	M:C = 40:25 (μm)^b^	M:C = 23:23 (μm)^d^	M:C = 20:20 (μm)	?	almost the same diameter between M and C (Fig 2D)	M:C = 55:24 (μm)	M:C = (8–23):14 (μm)	?
**Pharynx length (mm)**	4.5–5 (ca. 1/3 body length)^b^	12.5 (ca. 1/2 body length)^d^	?	? (ca. 1/3 body length)	1.2	0.5–1.1 (ca. 1/4 body length)	2.6–6.6 (ca. 1/3 body length)	?
**Prostatoid organ number**	25^a^; <30^b^	>50^d^	ca. 40	10–18	16–24	2–4	21–22	3, 7
**Girdle**	triple^a, c^	triple^e^	double	single	single	single	single	single
**Uterine vesicle number**	several^b^	8–10 in each^d^	5 in each	several	?	2 in each	5 in each	?
**Inner wall of Lang’s vesicle**	non-ciliated^c^	ciliated^e^	ciliated	partly ciliated	?	lined with cilia; elongated in posterior region	lined with cilia; elongated in posterior region	non-ciliated
**Connection point of uterine canals**	vagina immediately anterior to the entrance of Lang’s vesicle^b^	vagina immediately anterior to the entrance of Lang’s vesicle^d^	vagina immediately anterior to the entrance of Lang’s vesicle	vagina at its junction with Lang’s vesicle	?	lateral to Lang’s vesicle	vagina immediately anterior to the entrance of Lang’s vesicle	?
**Habitat**	under stones at high water line; beach-rock^b^	under stones at highwater line^d^	under stones near the highwater limit	coarse sediments (shell and madrepore fragments)	under littoral to sublittoral rocks	interstitial coarse sand	under intertidal rocks	?
**Distribution**	Charotte Amalie, St. Thomas, Virgin Islands (USA)^a^; Curaçao^b^; Kralendijk, near Pasanggrahan, Bonaire^b^	Piscadera Baai, St. Michels Baai, Vaerssen Baai, Curaçao^d^	Haha-jima and Chichi-jima (Ogasawara Islands), Japan	Eilat, Israel (Red Sea); Akko, Shiqmona, Israel (Mediterranean Sea)	Agua Blanca, San Agustinillo, Panteón, Estacahuite, Dos Hermanas beaches, Cacaluta Bay, Oaxaca	Nagahama Beach, Okinawa Island, Japan	Nagahama Beach, Okinawa Island, Japan	Samboanga, Philippines
**Reference**	^a^[34] ^b^[35] ^c^[30]	^d^[35] ^e^[30]	[30, 31]	[30]	[36]	this study	this study	[30]

Further examination is required to evaluate whether *Boninia* sp. from Samboanga (the Philippine Islands) [30] is conspecific with the new species described here. *Boninia* sp. was collected from Samboanga and originally identified as *B*. *mirabilis* by Bock [31]. Later, Curini-Galletti & Campus [30] re-examined Bock’s [31] voucher specimens and recognized them as an undescribed species based on their internal morphology, including (*i*) the very small number (3–7) of prostatoid organs arranged into a single girdle and (*ii*) the completely unciliated Lang’s vesicle. The new species is similar to the specimens from Samboanga in terms of the small number of prostatoid organs and the single girdle, but it differs by its entirely ciliated Lang’s vesicle.

### *Boninia yambarensis* sp. nov.

urn:lsid:zoobank.org:act:7C61DFF6-51A3-469C-9274-069C7EBE2729

(Figs 4–6)

**Fig 4:**
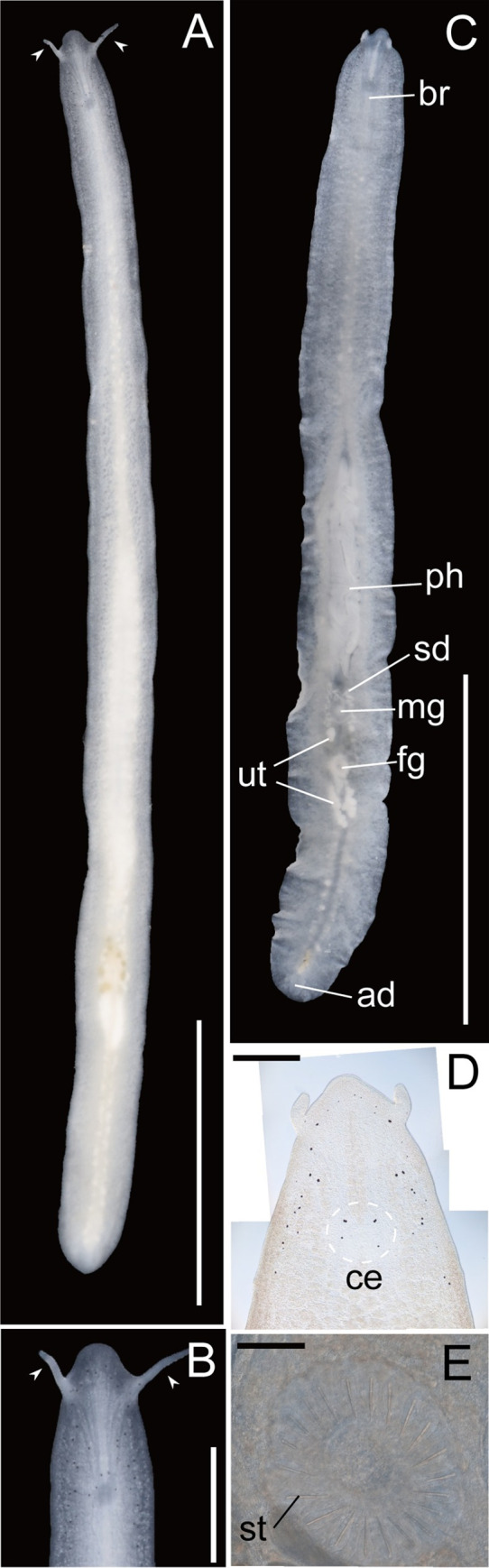
*Boninia yambarensis* sp. nov.: Photographs taken in the living state. (A) ICHUM 8284, dorsal view; (B) ICHUM 8284, magnification of the anterior body, dorsal view; (C) ICHUM 8288, ventral view; (D) ICHUM 8289, magnification of anterior body (squeezed); (E) ICHUM 8289, arrangement of prostatoid organs (squeezed). Pointed tentacles are shown by arrowheads (A, B). Abbreviations: ad, adhesive organ; br, brain; ce, cerebral eyespots; fg, female gonopore; mg, male gonopore; ph, pharynx; sd, sperm duct; st, stylet; ut, uteri. Scale bars: A, C, 5 mm; B, 1 mm; D, 500 μm; E, 300 μm.

**Fig 5:**
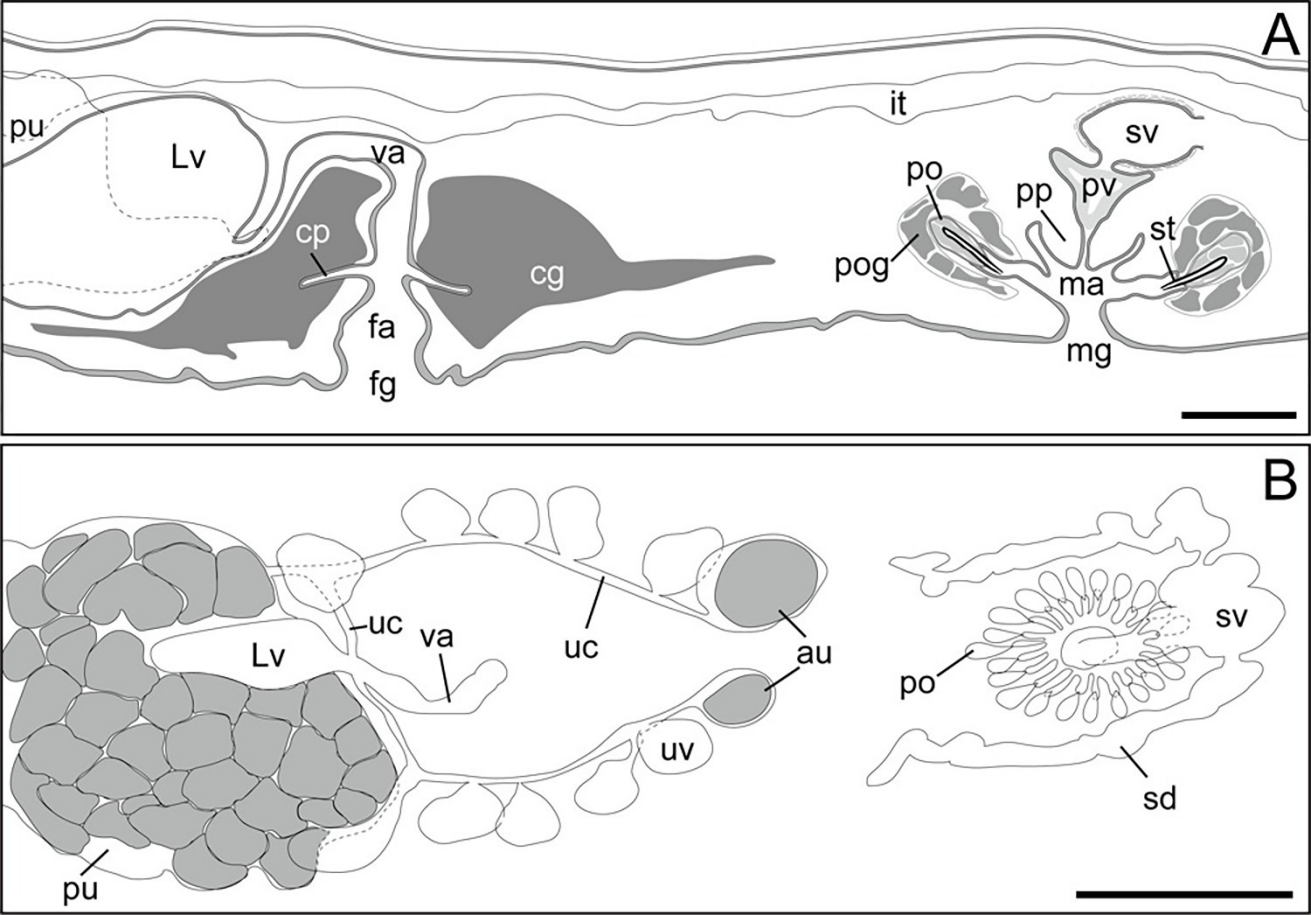
Schematic diagrams of *Boninia yambarensis* sp. nov. (anterior to the right). (A) ICHUM 8284 (holotype), sagittal view of copulatory complex; (B) ICHUM 8285 (paratype), histological view of copulatory complex. Abbreviations: au, anterior dilation of uterus; cg, cement glands; cp, cement pouch; fa, female atrium; fg, female gonopore; it, intestine; Lv, Lang’s vesicle; ma, male atrium; mg, male gonopore; po, prostatoid organ; pog, prostatoid organ glands; pp, penis papilla; pu, posterior dilation of uterus; pv, prostatoid vesicle; sd, sperm duct; st, stylet; sv, seminal vesicle; uc, uterine canal; uv, uterine vesicle; va, vagina. Scale bars: A, 100 μm; B, 300 μm.

**Fig 6:**
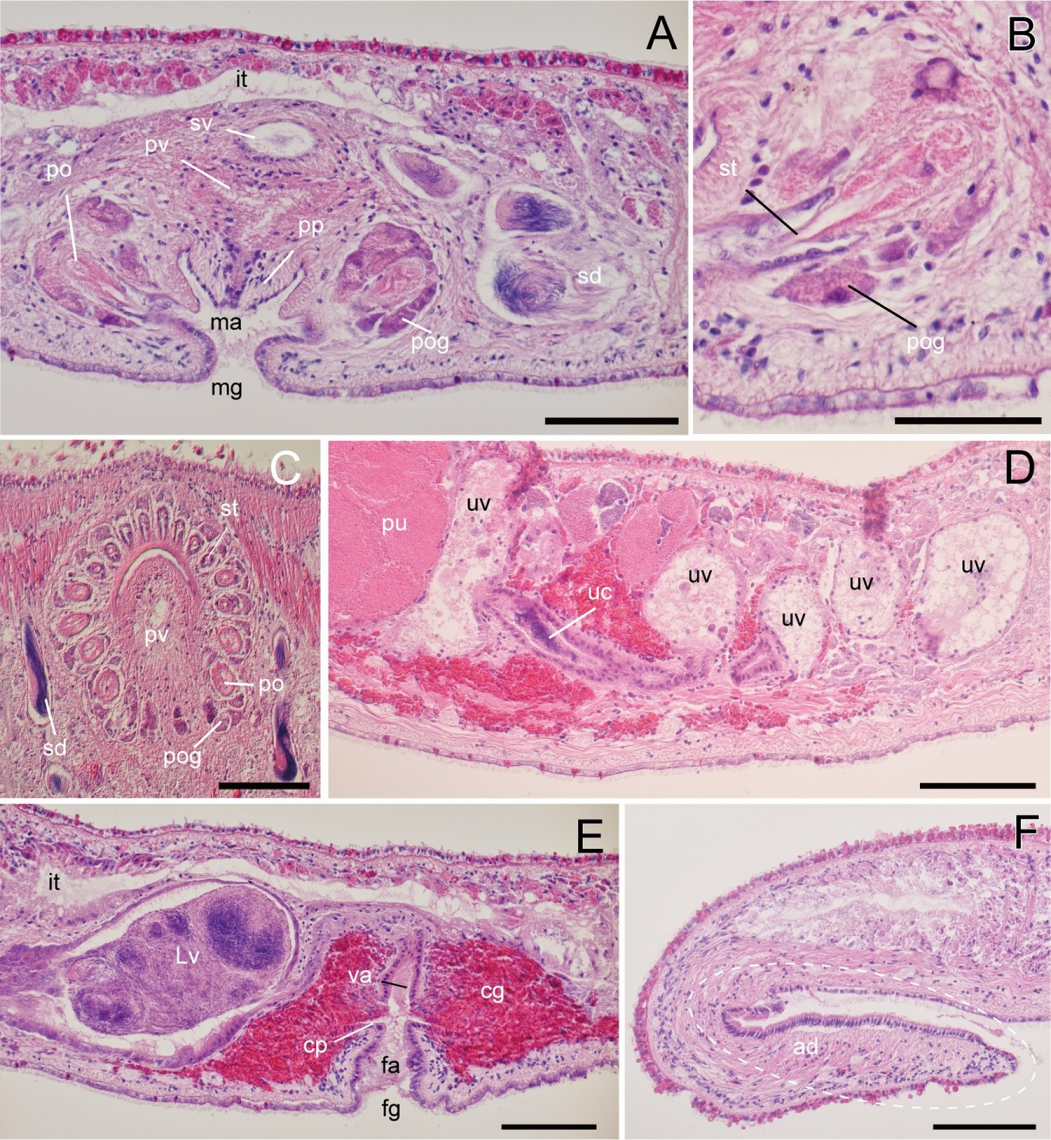
*Boninia yambarensis* sp. nov.: Photomicrographs of sagittal (A, B, D–G) and horizontal sections (C). (A) ICHUM 8284 (holotype), male copulatory apparatus, anterior to the right; (B) ICHUM 8284 (holotype), prostatoid organ, anterior to the right; (C) ICHUM 8285 (paratype), arrangement of prostatoid organs; (D) ICHUM 8284 (holotype), uterine vesicles, anterior to the right; (E) ICHUM 8284 (holotype), female copulatory apparatus, anterior to the right; (F) ICHUM 8284 (holotype), adhesive organ. Abbreviations: ad, adhesive organ; au, anterior dilation of uterus; cg, cement glands; cp, cement pouch; fa, female atrium; fg, female gonopore; it, intestine; Lv, Lang’s vesicle; ma, male atrium; mg, male gonopore; po, prostatoid organ; pog, prostatoid organ glands; pp, penis papilla; pu, posterior dilation of uterus; pv, prostatoid vesicle; sd, sperm duct; st, stylet; sv, seminal vesicle; uc, uterine canal; uv, uterine vesicle; va, vagina. Scale bars: A, C–F, 100 μm; B, 50 μm.

#### Material examined

*Holotype*. ICHUM 8284, sagittal sections of the posterior body (HE; nine slides) along with the remaining unsectioned body (preserved in 70% ethanol); Nagahama Beach, Okinawa, Japan (26.6242°N, 128.1843°E); coll. A. Tsuyuki, August 9, 2021.

*Paratypes*. Five specimens (collection site and collector same as holotype). ICHUM 8285, horizontal sections of the posterior body (HE; three slides) along with the remaining unsectioned body (preserved in 70% ethanol); March 31, 2021. ICHUM 8286, sagittal sections of the posterior body (HE; six slides) along with the remaining unsectioned body (preserved in 70% ethanol); March 31, 2021. ICHUM 8287, sagittal sections of the posterior body (MT; eight slides) along with the remaining unsectioned body (preserved in 70% ethanol); March 31, 2021. ICHUM 8288, unsectioned, preserved in 70% ethanol; August 9, 2021. ICHUM 8289, unsectioned, preserved in 10% formaldehyde solution; March 31, 2021.

#### Etymology

The new species is named after the region Yambaru, the northern part of Okinawa Island. The type locality, Nagahama Beach, is located in the southeastern Yambaru region.

#### Diagnosis

Body narrow and elongated; pair of pointed tentacles located at anterolateral margins; 3–4 pairs of cerebral eyespots and 19–42 marginal eyespots; 21–22 prostatoid organs arranged into single girdle; five uterine vesicles present in each oviduct; Lang’s vesicle fully ciliated; subepidermal muscle fibers of adhesive area not well developed.

#### Description

Body slender and elongated, tapered posteriorly, 13.9–22.4-mm long (22.4 mm in holotype) and 0.93–1.25-mm wide (1.25 mm in holotype) in living state (Fig 4A). Pair of pointed tentacles located at sides of head (Fig 4A and 4B), 0.3–0.6-mm long (0.63 mm in holotype). Dorsal surface smooth, translucent, without any coloration (Fig 4A). Ventral surface translucent (Fig 4C).

Tentacular eyespots absent. Pair of 3–4 cerebral eyespots present (ca. 14 μm in diameter); in each part of pair, two eyespots lying close to each other with one or two eyespot(s) located at distance of about 0.3 mm posterior to frontal two eyespots (Fig 4D). Marginal eyespots (ca. 8–23 μm in diameter), 19–42 in number (35 in holotype), distributed anteriorly along margins on both sides (Fig 4B and 4D).

Intestine highly branched, spreading all over body. Pharynx ruffled, 2.6–6.6-mm long (6.6 mm in holotype), lying on body center (Fig 4C). Mouth situated in center of pharynx.

Male gonopore situated immediately behind posterior end of pharynx (Fig 4C). Male copulatory apparatus consisting of seminal vesicle, interpolated prostatoid vesicle, penis papilla, and 21–22 prostatoid organs (Figs 4E, 5A, and 5B). Pair of sperm ducts running on each side of midline, curving at posterior position of pharyngeal pouch to separately enter into seminal vesicle (Figs 4C and 5B). Seminal vesicle spherical, coated with thin muscle fibers, distally opening into prostatoid vesicle (Figs 5A and 6A). Prostatoid vesicle, 44-μm long in dorsoventral axis, lined with high epithelium, connecting to penis papilla (Figs 5A and 6A). Penis papilla unarmed, 45-μm long in dorsoventral axis, projecting into male atrium (Figs 5A and 6A). Inner wall of male atrium well ciliated (Fig 6B). Individual ducts of 21–22 prostatoid organs (21 in holotype) radially arranged into single girdle around male atrium and prostatoid vesicle (Figs 4E, 5B, and 6C), opened into inner area of male atrium. Each prostatoid organ oval in shape, about 21-μm long in its longest axis, bearing sclerotized stylet (53 μm in length) (Figs 5A, 6A and 6B). Extracapsular glands (“prostatoid organ glands”) producing glandular secretion into each prostatoid organ (Figs 5A, 6A and 6B).

Pair of uterine canals running on both sides of midline, connecting to vagina immediately anterior to entrance of Lang’s vesicle (Fig 4D); each canal connected through short side branches to five uterine vesicles (Figs 5B and 6D). Each uterine canal forming uteri at most anterior and posterior dilations; uteri filled with eggs (Figs 5D and 6D). Lang’s vesicle elongated (340 μm in its long axis; 219 μm in its short axis); inner wall lined with cilia; elongated cilia observed in posterior region (Figs 5A, 5B and 6E). Vagina lined with cilia, curving down and leading to cement pouch (Figs 5A and 6E). Cement glands numerous, concentrated around female copulatory apparatus and releasing contents into cement pouch (Fig 6E). Female atrium opening to exterior through female gonopore.

Epidermis on dorsal side ciliated, with numerous ovoid rhabdites (Fig 6A and 6E). Ventral epidermis ciliated except for adhesive area (Fig 6F).

Adhesive organ located at posterior end of body on ventral side (Figs 4C and 6F). Subepidermal muscle fibers not well developed in adhesive area.

#### Distribution

The species is known from the type locality, Nagahama Beach, eastern coast of Okinawa Island, Japan.

#### Habitat

To date, confirmed only from under rocks in the intertidal region. The thin body width (1–2 mm) of this species suggests that it may also be able to inhabit intergravel spaces. However, *B*. *yambarensis* sp. nov. seems to have a preference for epibenthic habitats over interstitial habitats because (*i*) it has yet to be collected from interstitial environments and (*ii*) more than 10 individuals of the species were found under rock surfaces independently in our two surveys.

#### Remarks

The materials examined belong to *Boninia* because they conform to the generic diagnosis, i.e., they have two or more prostatoid organs with stylets opening into the inner area of the male atrium. *Boninia yambarensis* sp. nov. can be separated from *B*. *antillara*, *B*. *divae*, and *B*. *mirabilis* by its single girdle of prostatoid organs [30, 31]. *Boninia yambarensis* sp. nov. resembles *B*. *neotethydis*, *B*. *oaxaquensis*, and *B*. *uru* sp. nov. in having a single girdle of prostatoid organs (Table 4); however, it can be distinguished from *B*. *neotethydis* and *B*. *uru* sp. nov. by the number of prostatoid organs (21–22 in *B*. *yambarensis* sp. nov.; 10–18 in *B*. *neotethydis*; and 2–4 in *B*. *uru* sp. nov.). The number of prostatoid organs are the same in *B*. *yambarensis* sp. nov. and *B*. *oaxaquensis*; however, the new species can be discriminated from *B*. *oaxaquensis* by the number of cerebral eyespots (6–7 in *B*. *yambarensis* sp. nov.; 14–66 in *B*. *oaxaquensis*).

### Molecular phylogeny

**Fig 7:**
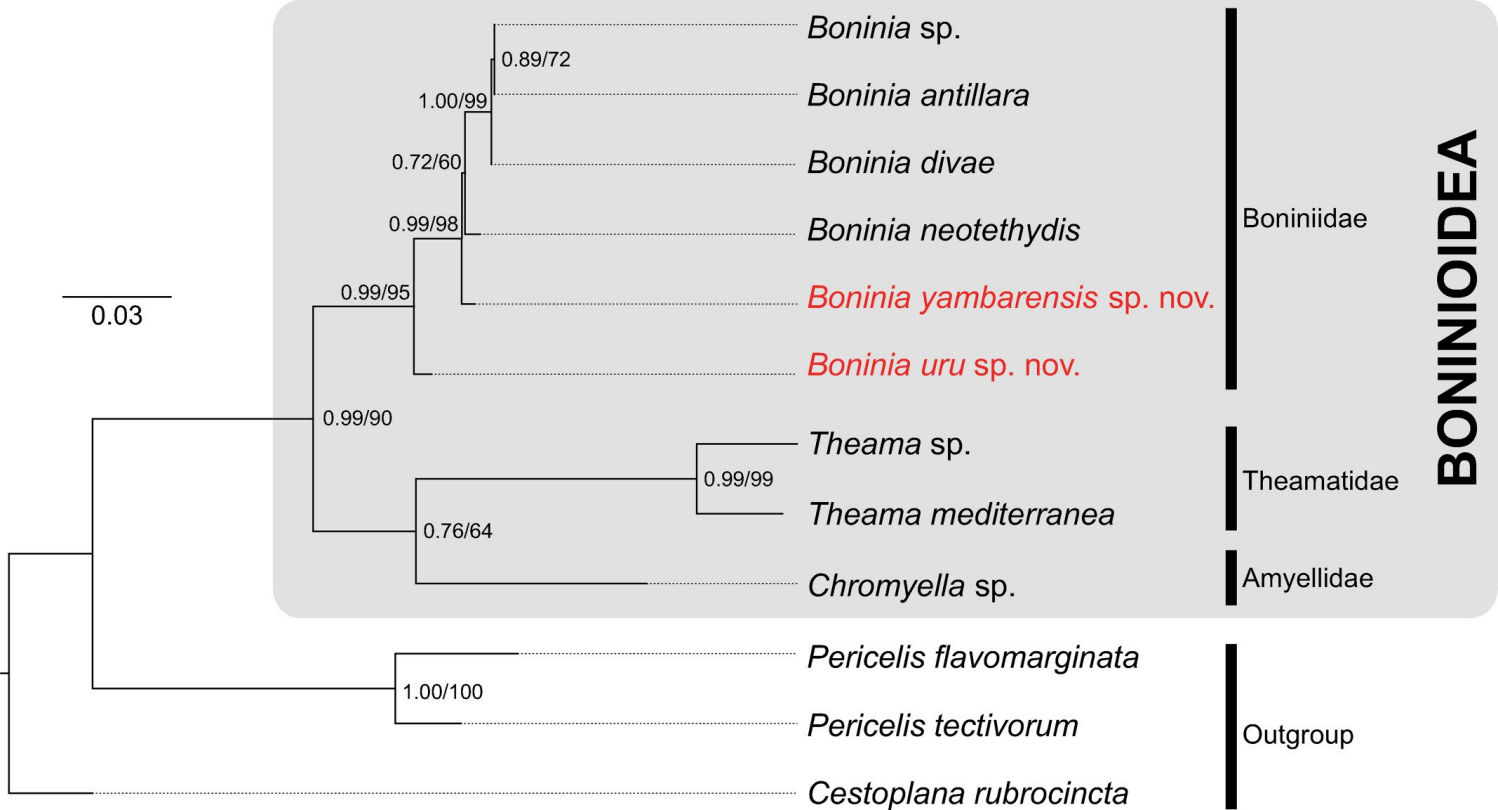
Maximum likelihood phylogenetic tree based on a concatenated dataset of partial 18S and 28S. Numbers near nodes are posterior probability and bootstrap values, respectively.

The resulting BI and ML trees were identical in terms of topology; all six species of *Boninia* exclusively formed a clade (0.99 PP; 95% BS) (Fig 7). *Boninia yambarensis* sp. nov. formed a clade with *B*. *antillara*, *B*. *divae*, *B*. *neotethydis*, and *Boninia* sp. with high support (0.99 PP; 98% BS). *Boninia antillara*, *B*. *divae*, and *Boninia* sp. were monophyletic with high support (1.00 PP; 99% BS). However, the phylogenetic relationship among Boniniidae (represented by the six species), Theamatidae (represented by *Theama mediterranea* and *Theama* sp. of Laumer & Giribet [54]), and Amyellidae (represented by *Chromyella* sp. of Laumer & Giribet [54]) remains unclear due to low support values (0.76 PP; 64% BS) (Fig 7).

### Ancestral habitats

**Fig 8:**
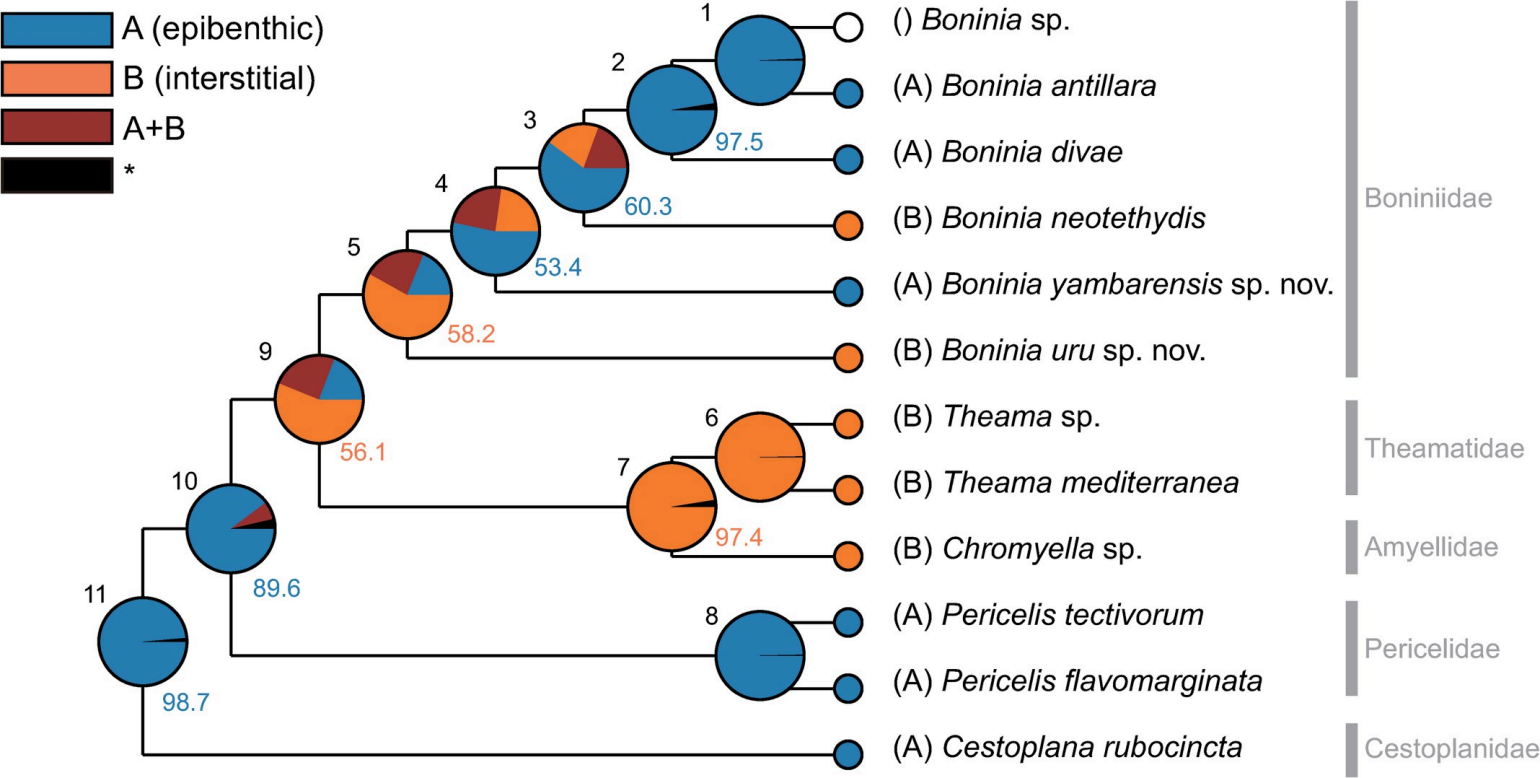
Ancestral reconstruction of habitats produced using Bayesian Binary Markov chain Monte Carlo analysis. Nodes are numbered. Pie charts on nodes show the probabilities of possible ancestral states with numbers representing the highest probabilities (%). *, ancestral state with a relative probability <5%.

The ancestral states of habitats reconstructed via BBM analysis are shown in Fig 8. The last common ancestor (LCA) of all analyzed species, including the outgroups (node 11), was estimated to be epibenthic with a probability of 98.7%. The LCAs of Boninioidea sensu Dittmann et al. [41] (node 9) and Boniniidae (node 5) appeared to be interstitial, although the estimated probabilities were relatively low (56.1% and 58.2%, respectively). In contrast, the LCA of *Boninia* sp., *B*. *antillara*, and *B*. *divae* (node 2) and that of *Boninia* sp. and *B*. *antillara* (node 1) were epibenthic with high probabilities of 97.5% and 99.8%, respectively. Also, the ancestral states of nodes 3 and 4 were likely to be epibenthic, which was the most favored state (53.4% and 60.3%, respectively).

### Discussion

Our phylogenetic results suggest the possibility that an unexpected evolutionary scenario occurred in the *Boninia* lineage. *Boninia uru* sp. nov. was sister to a clade composed of the remaining five congeners, in contrast to both our stated hypotheses (see the Introduction), in which this new species would have been sister to the interstitial *B*. *neotethydis* (“interstitial monophyly hypothesis”) or to the sympatric *B*. *yambarensis* (“*in situ* speciation hypothesis”). Thus, in this section and based on our results, we discuss the most plausible evolutionary hypotheses pertaining to (*i*) the shift between interstitial and noninterstitial microhabitats and (*ii*) the settlement of the two species at the same beach.

### Reversible evolutionary shifts from interstitial to epibenthic realms in the *Boninia* lineage

The results of our ancestral state reconstruction analysis show that early boniniids likely lived in interstitial microhabitats, with some descendants subsequently having evolved to inhabit epibenthic environments, whereas others either remained in (Fig 8). The LCA of all analyzed *Boninia* species (node 5) was estimated as interstitial, although this estimation is not supported with high probability (58.2%) (Fig 8). In contrast, the LCA of *Boninia* sp., *B*. *antillara*, and *B*. *divae* (node 2) and that of the former two species (node 1) were estimated to be epibenthic with high support (97.5% and 99.8%, respectively). These results suggest an evolutionary scenario in which the LCA of all analyzed *Boninia* species inhabited an interstitial environment, and where the LCA of *Boninia* sp., *B*. *antillara*, and *B*. *divae* subsequently changed to an epibenthic lifestyle.

A prerequisite for this interpretation is that microhabitat preference of adults is species-specific and alternative, i.e., boniniids in the same species do not occur simultaneously in both interstitial and noninterstitial environments at random in their mature state. We consider this assumption to be realistic and applicable based on our observations. In our three independent field surveys, we collected six individuals of *B*. *uru* sp. nov. only by washing gravel sediments near the highwater limit where rocks were absent. In contrast, on the same beach, we observed >10 individuals of *B*. *yambarensis* sp. nov. crawling on undersurfaces of rocks in the lower intertidal zone. These observations indicate a narrow habitat range at least for each new species described herein (see also habitat for *B*. *yambarensis* above). Such a habitat preference would be expected for the other analyzed species *B*. *antillara*, *B*. *divae*, and *B*. *neotethydis* by extrapolating the empirical evidence observed in our two new species, although the actual microhabitat for each of the other congeners should be confirmed in additional investigations in the future.

The evolutionary shift from interstitial to noninterstitial habitats is likely uncommon among Animalia. Indeed, irreversible one-way transition from the noninterstitial realm to the interstitial realm seems to be the norm among metazoan taxa investigated to date; such interstitial taxa are exclusively monophyletic, e.g., Dinophilidae, Diurodrilidae, Polygordidae, Protodrilidae, Psammodrilidae [60] (Annelida), Ototyphlonemertidae [61, 62] (Nemertea), and Rhodopemorpha [63] (Mollusca), with the notable exception of acochlidean slugs in the clade Hedylopsacea [15]. Moreover, even among acochlidian slugs, evolutionary transitions from interstitial to noninterstitial habitats are limited to species living in specialized habitats, such as those exposed to nonmarine salinities (brackish, limnic, and amphibious species) [15] and living in the deep sea [64], whereas almost all other species of acochlidian slugs live in shallow waters. Our study suggests a habitat shift from the interstitial to noninterstitial marine realm in the evolutionary history of flatworms based on molecular phylogenetic evidence with statistical support.

It remains unclear what makes such unique evolutionary transitions from interstitial to noninterstitial habitats possible in the *Boninia* lineage. The relatively high phenotypic plasticity in adult body size (about >2–10 times) among polyclads [cf. 65] might be related to the evolutionary pathway. As Westheide [4] stated, body size is one of the most important factors for microhabitat shifts between interstitial and noninterstitial realms. In acochlidians, “secondary gigantism” in body size (see [4, 66]) may have contributed to the evolutionary shift from interstitial to epibenthic habitats; secondary gigantism is likely to be a consequence of adaptation to brackish water, freshwater, and terrestrial systems [15] or to limitations of food resources in the deep sea [64]. If interstitial boniniids show plasticity in body size, accidental “gigantism” could potentially have led to a lifestyle outside interstitial biotopes, similar to the known example in acochlidians.

### Independent colonization of the same beach

Our tree topology suggests that *B*. *uru* sp. nov. and *B*. *yambarensis* sp. nov. settled at the same beach independently. In the resulting tree, *B*. *yambarensis* sp. nov. was more closely related to the Caribbean and Lessepsian species (*B*. *antillara*, *B*. *divae*, *B*. *neotethydis*, and *Boninia* sp. of Laumer & Giribet [54]) than to the sympatric species *B*. *uru* sp. nov. (Fig 7). Additionally, the two new species clearly differ morphologically in their reproductive organs, i.e., the number of prostatoid organs (2–4 in *B*. *uru* sp. nov.; 21–22 in *B*. *yambarensis* sp. nov.). Thus, there seems to be deep divergence between the two new species, and they may have encountered the collection site after they had been reproductively isolated.
